# Obstetric anaesthesia for caesarean section in Central Europe: a prospective population-weighted binational audit (OBAAMA-COV Study)

**DOI:** 10.1186/s44158-026-00435-2

**Published:** 2026-07-08

**Authors:** Jan Bláha, Pavlína Nosková, Dagmar Seidlová, Monika Grochová, Radka Klozová, Josef Firment, Hana Harazim, Kateřina Kusalová, Michal Svoboda, Deana Slovjaková, Nela Rezbáriková, Quynh Giang Nguyenová, Petr Štourač, the OBAAMA-C. O. V. Study Group, Miriama Kaščáková, Klára Fabiánková, Jaroslava Ščamburová, Petr Reimer, Monika Burešová, Richard Tesařík, Pavel Kettner, Xénia Silová, Christian Kufa, Marie Chaloupecká, Nikola Bandíková, Alena Baničová, Libor Machát, Tomáš Čechmánek, Pavel Longin, Pavel Neumann, Jana Mahdalová, Tomáš Piksa, Terézia Klenová, Jiří Pařízek, Hana Pachovská, David Míra, Kristýna Kalinová, Edita Langrová, Jan Němec, Marián Olejník, Michal Houfek, Petra Běhounková, Jana Daňová, Marek Proksa, Veronika Richterová, Petr Štěpánek, Jana Popieluchová, Jana Pěkná, Ladislav Rychtárik, Lucie Chamrová, Josef Kubík, Gabriela Pohořelá, Jakub Bala, Martin Hudymač, Jaroslava Márová, Monika Ďuricová, Prokop Seif, Jiří Cetkovský, Radek Majerčin, Zdeněk Kos, Radovan Prchlík, Jaroslav Čupera, Kazimír Kovář, Dušana Bednářová, Martin Dvořák, Petr Vašíček, Marek Spyra, Soňa Fajstauerová, Pavel Kopecký, Ivo Schindler, Marek Zbořil, Martin Šimák, Eva Smržová, Vladislav Černý, Lukáš Rokos, Milan Náprstek, Daniel Kmec, Petr Kozár, Vladimír Zemánek, Igor Krupa, Petr Kudrna, Martina Bělorová, Mária Petrisková, Jana Morávková, Jakub Vallo, Hana Malérová, Renáta Toboláková, Matúš Pauliny, Dária Rábarová, Ľubomíra Romanová, Michal Blanár, Jana Buranská, Katarína Galková, Jana Šimonová, Božena Horanová, Michal Venglarčík, Ľuboš Filiač, Stanislava Richterová, Adam Antolík, Marek Pastir, Dušan Kostovčík, Sharifullah Azizi, Mária Šramková, Ľubica Mišáková, Serhiy Rak, Ivo Horský, Zuzana Baluchová, Dávid Druska, Lucia Cifraničová, Eva Fašánková, Ľuba Jurčíková, Stanislav Saladiak, Ivana Ivanová

**Affiliations:** 1 Department of Anaesthesiology and Intensive Care Medicine, First Faculty of Medicine, Charles University and General University Hospital in Prague, Prague, Czech Republic; 2 Anaesthesiology and Resuscitation Department II, University Hospital Brno, Brno, Czech Republic; 3 1st Department of Anaesthesiology and Intensive Medicine, Pavol Jozef Šafárik University, Faculty of Medicine and Louis Pasteur University Hospital, Košice, Slovakia; 4 Department of Anaesthesiology and Intensive Care Medicine, Motol and Homolka University Hospital, Prague, Czech Republic; 5 Department of Paediatric Anaesthesiology and Resuscitation, Medical Faculty of Masaryk University and University Hospital Brno, Brno, Czech Republic; 6 Institute of Biostatistics and Analyses, Ltd., Brno, Czech Republic; 7 Department of Anaesthesiology and Intensive Care Medicine, Medical Faculty of Masaryk University and University Hospital Brno, Brno, Czech Republic; 8 Department of Simulation Medicine, Medical Faculty of Masaryk University, Brno, Czech Republic

**Keywords:** Caesarean section, General anaesthesia, Neuraxial anaesthesia, Obstetric anaesthesia, Maternal preference, Central Europe, Clinical audit

## Abstract

**Background:**

Neuraxial anaesthesia is recommended as the preferred technique for caesarean section, and the proportion performed under general anaesthesia is increasingly regarded as a quality indicator. Previous national audits in the Czech Republic and Slovakia demonstrated a reduction in general anaesthesia rates between 2011 and 2015; however, contemporary population-level data are lacking.

**Methods:**

OBAAMA-COV was a prospective, cross-sectional national audit conducted in November 2022 across maternity units in the Czech Republic and Slovakia. Consecutive caesarean sections were recorded using a structured electronic registry. Population weighting based on official national statistics was applied to generate representative estimates of anaesthetic technique distributions and reported indications. The analyses were descriptive.

**Results:**

A total of 2,270 caesarean sections were recorded from 94 centres. Population-weighted estimates corresponded to 2,068 procedures in the Czech Republic and 1,205 in Slovakia. Overall general anaesthesia rates were 33.9% and 31.3%, respectively. In elective procedures, general anaesthesia was used in 23.0% (95% CI 19.9–26.4) in the Czech Republic and 24.9% (20.6–29.7) in Slovakia; in emergency procedures, rates were 45.4% (42.1–48.8) and 36.8% (31.8–42.1), respectively. Maternal preference was the leading reported indication in elective cases (approximately 60–63% across audit cycles), whereas urgency predominated in emergency settings. Rapid sequence induction with tracheal intubation was used in most cases; difficult intubation occurred in approximately 2% of cases, and no aspiration was recorded. Umbilical arterial pH values were comparable between general anaesthesia and neuraxial techniques.

**Conclusions:**

These population-weighted data demonstrate a persistent divergence from contemporary European standards. General anaesthesia for caesarean section remains common in both countries, plateauing rather than declining since 2015. The continued reliance on general anaesthesia—driven by maternal preference in elective procedures and by urgency in acute settings—suggests an important contribution of organisational and sociocultural factors beyond strictly clinical indications.

**Trial registration:**

ClinicalTrials.gov (NCT04912791), registered June 2, 2021.

**Supplementary Information:**

The online version contains supplementary material available at https://doi.org/10.1186/s44158-026-00435-2.

## Background

Caesarean section is the most frequently performed major surgical procedure worldwide, and its incidence continues to rise across Europe [1, 2]. International guidelines and European consensus statements uniformly recommend neuraxial anaesthesia as the preferred technique for both elective and emergency caesarean section, primarily to reduce airway-related complications and optimise maternal safety [3, 4]. Consequently, the proportion of caesarean sections performed under general anaesthesia (GA) is increasingly regarded as a system-level quality indicator in obstetric anaesthesia [4, 5], with several Western European countries reporting elective general anaesthesia rates below 1% [5].

In Central Europe, previous national audits conducted in the Czech Republic and Slovakia (OBAAMA 2011 and 2015) documented substantially higher general anaesthesia rates compared with Western European benchmarks [6, 7]. A marked reduction in the use of general anaesthesia was observed between 2011 and 2015. However, without contemporary population-level data, it is unclear whether this positive trend has continued, and what specific clinical or organisational factors currently drive the ongoing use of general anaesthesia in elective and emergency settings.

The OBAAMA-COV project was designed as a prospective, population-weighted national audit of obstetric anaesthetic practice. Following publication of the labour analgesia component [8], the present analysis focuses on anaesthesia for caesarean section. The primary objective was to describe current practice in the Czech Republic and Slovakia, using population weighting to generate representative national estimates [9]. Secondary objectives were to compare elective and emergency procedures, to analyse reported indications for general anaesthesia—including maternal preference—and to determine whether previously observed improvements have successfully closed the gap with current European standards.

## Methods

### Study design and ethical approval

OBAAMA-COV was a prospective, cross-sectional national audit of obstetric anaesthesia practice conducted in the Czech Republic and Slovakia between 1 and 30 November 2022. It represents the third phase of the OBAAMA programme, following national audits performed in 2011 and 2015 [6, 7]. The “COV” phase reflects continuation of the programme during the COVID-19 era; however, the study was not designed to assess pandemic-related effects on anaesthetic practice.

In the Czech Republic, the study was approved by the Ethics Committee of University Hospital Brno, and informed consent was waived. In Slovakia, approval was granted by the Ethics Committee of Louis Pasteur University Hospital, Košice, and written informed consent was obtained from the participants.

### Setting and participants

All maternity units in both countries were invited to participate, including university, regional, and local hospitals. In Slovakia, centres are formally classified into levels I–III + according to the complexity of perinatal care. Each participating centre prospectively recorded all consecutive caesarean sections performed during the study period. The audit was purely observational and did not influence clinical management.

### Centre recruitment

All maternity units were invited via emails to the respective heads of department. Non-responding centres received targeted follow-up emails. In cases of continued non-participation, members of the steering committee contacted the departmental heads personally by telephone. The project was formally endorsed by the Czech Society for Anaesthesiology, Resuscitation, and Intensive Care Medicine (CSARIM) and the Slovak Society of Anaesthesiology and Intensive Medicine (SSAIM).

### Data collection

Data were collected using a structured web-based electronic case report form (CLADE-IS, Masaryk University, Czech Republic). The registry captured centre-level characteristics and detailed procedural data.

Data completeness and internal consistency were verified prior to analysis. Mandatory fields and logical range checks were implemented within the electronic case report form to minimise missing or implausible entries. The database structure was aligned with previous OBAAMA audits to ensure longitudinal comparability [6, 7].

Indications for general anaesthesia were selected from predefined registry categories and reported prospectively by the attending anaesthesiologist at the time of the procedure; no external adjudication was performed.

### Classification of urgency

Caesarean sections were classified as elective or emergency. The emergency category comprised Lucas' urgency categories 1–3 [10, 11]. These categories were analysed as a single aggregate group to preserve longitudinal comparability with previous OBAAMA audits. Furthermore, this approach minimises inter-centre classification bias, given the absence of a uniform national triage system in either country. The study was not designed to evaluate urgency-specific performance indicators or decision-to-delivery intervals.

### Representativeness and population weighting

Participation rates varied across centre types. To obtain nationally representative estimates, population weighting was applied using official national statistics from the Institute of Health Information and Statistics of the Czech Republic (ÚZIS) and the National Health Information Centre of Slovakia (NCZI). Centres were weighted by maternity centre type and the corresponding national caesarean section volumes. Population weighting was applied exclusively to national estimates of anaesthetic technique distribution and reported reasons for GA. Detailed procedural characteristics are presented as unweighted registry data to reflect actual reported practice within participating centres.

### Statistical analysis

All analyses were descriptive. Categorical variables are presented as frequencies and percentages with 95% confidence intervals (CI). Continuous variables are reported as medians with interquartile ranges (IQR). Confidence intervals reflect the precision of population-weighted estimates and were not used for formal hypothesis testing. For strata with complete national coverage (100% participation), applying the finite population correction yielded zero sampling variance for specific estimates, resulting in identical lower and upper confidence interval bounds. No formal hypothesis testing or between-country comparisons were performed. Analyses were performed using R (R Foundation for Statistical Computing, Vienna, Austria). LLM-assisted tools were used for language editing. The authors reviewed, edited, and approved all text and take full responsibility for its content.

## Results

### Study population and national representativeness

A total of 94 maternity centres participated in the audit: 67 of 87 in the Czech Republic (12/12 university, 6/15 regional, and 49/60 local centres) and 27 of 53 in Slovakia (3/6 level III +, 7/8 level III, 10/18 level II, and 7/21 level I). Participating centres recorded 1,581 caesarean sections in the Czech Republic and 689 in Slovakia during the study period.

This corresponded to 76.5% of the national caesarean section volume in the Czech Republic and 57.2% in Slovakia between 1 and 30 November 2022.

Median maternal age was 32 years (IQR 28–35) in the Czech Republic and 31 years (IQR 27–35) in Slovakia. Median BMI was 29.4 kg/m^2^ and 28.7 kg/m^2^, respectively. Detailed maternal and obstetric characteristics are presented in Additional file 1: Table S2.

### Anaesthetic techniques for caesarean section

The distribution of anaesthetic techniques for elective and emergency caesarean sections is presented in Table 1. After population weighting, the analysis set comprised 2068 procedures for the Czech Republic and 1205 for Slovakia.

**Table 1:** Population-weighted estimates of anaesthetic techniques for caesarean section in the Czech Republic and Slovakia

	Czech Republic		Slovakia	
Number of births	7749*		3490**	
Number of caesarean deliveries	2068 (26.7%)*		1205 (34.5%)**	
General anaesthesia	33.9%		31.3%	
Neuraxial anaesthesia	66.1%		68.7%	
	Elective CS	Emergency CS	Elective CS	Emergency CS
	*N* = 940*	*N* = 1128*	*N* = 606**	*N* = 599**
General anaesthesia	23.0% (19.9–26.4)	45.4% (42.1–48.8)	24.9% (20.6–29.7)	36.8% (31.8–42.1)
*Neuraxial anaesthesia*	77.0% (73.6–80.1)	54.6% (51.2–57.9)	75.1% (70.3–79.4)	63.2% (57.9–68.2)
Spinal anaesthesia	93.9% (92.3–95.2)	74.0% (71.3–76.5)	97.8% (96.1–99.4)	85.3% (83.5–87.7)
Epidural top-up anaesthesia	0	19.9% (17.7–22.4)	0	13.8% (11.9–16.0)
Epidural ”de novo“ anaesthesia	6.1% (4.8–8.0)	6.1% (4.7–7.7)	1.1% (0.6–1.9)	0.5% (0.3–1.2)
CSE	0	0	1.1% (0.4–3.3)	0.4% (0.1–3.0)

Data are given in *n* (%) or percentages (95% CI). Data represent the final anaesthetic technique used for caesarean section. Cases in which neuraxial anaesthesia was attempted but required conversion to general anaesthesia were classified according to the final general anaesthetic technique; failed neuraxial blockade was recorded separately as a reported reason for general anaesthesia in Table 2. The percentages for neuraxial subtypes are conditional on neuraxial anaesthesia. Overall proportions reflect population-weighted estimates across all caesarean sections and are not simple averages of elective and emergency categories. National numbers of caesarean deliveries were derived from official statistics of the Institute of Health Information of the Czech Republic (*) and the National Health Care Information Centre of Slovakia (**). Emergency caesarean deliveries include urgency categories 1–3 according to the widely used Lucas classification [10]. Epidural top-up refers to the extension of labour epidural analgesia to anaesthesia for caesarean section; epidural “de novo” refers to newly administered epidural anaesthesia. *CI* confidence interval, *CS* caesarean section, *CSE* combined spinal–epidural anaesthesia

Neuraxial anaesthesia predominated in both countries; however, general anaesthesia accounted for approximately one-third of all caesarean sections overall and approximately one-quarter of elective procedures.

### Longitudinal comparison with previous OBAAMA audits

**Fig. 1:**
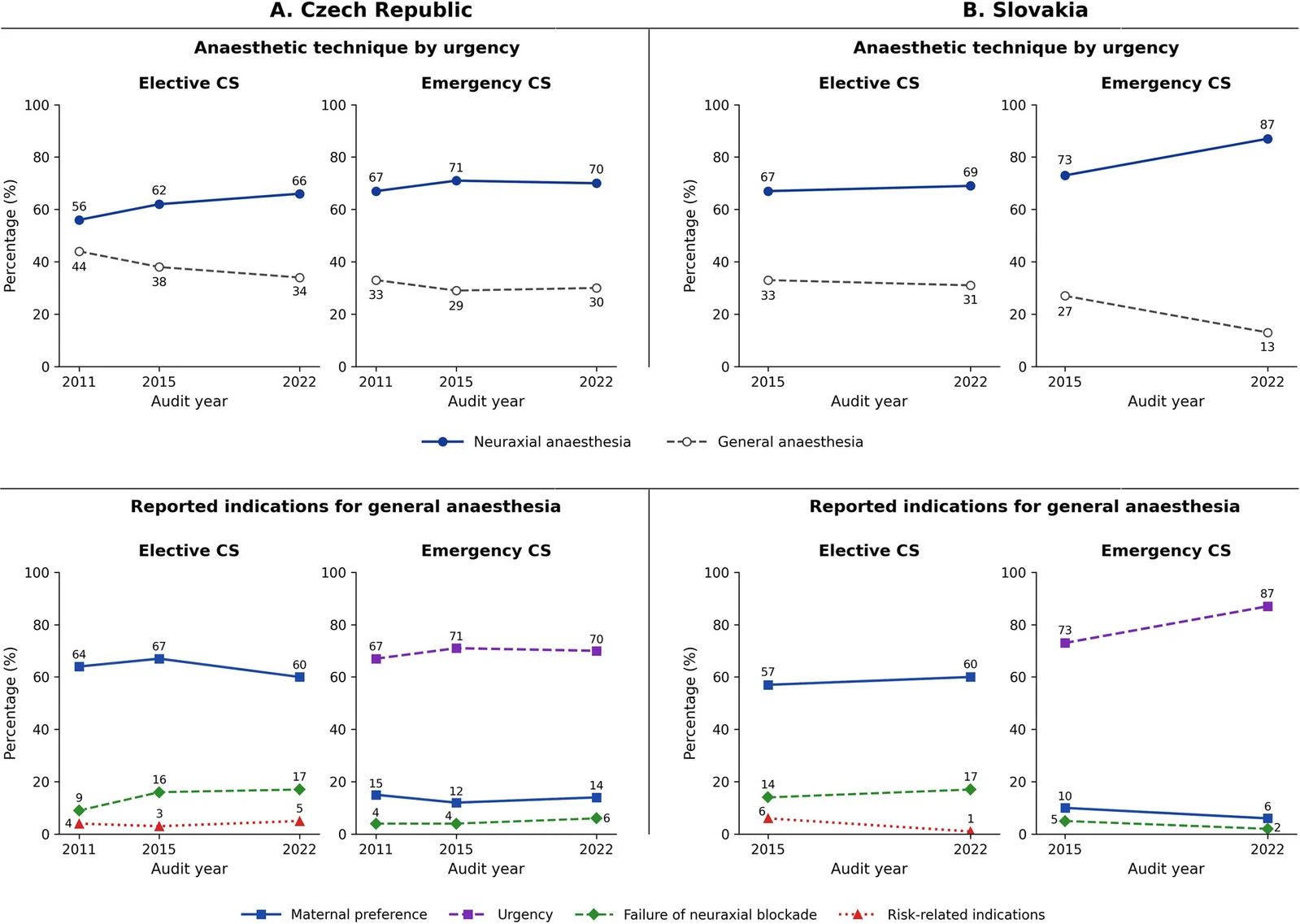
Temporal trends in anaesthetic technique and reported indications for general anaesthesia for caesarean section in the Czech Republic and Slovakia across OBAAMA audit cycles (2011–2022). Values are percentages. Data from the 2011 and 2015 OBAAMA audits were derived from previously published national surveys [6, 7]; 2022 data are from the present OBAAMA-COV audit. Emergency caesarean sections include Lucas categories 1–3 [10]. Indications for general anaesthesia were reported by the attending anaesthesiologist. For each country and urgency category, the three most frequently reported indications for general anaesthesia are shown. For elective caesarean sections, risk-related indications combine low-molecular-weight heparin use and placentation disorder

Temporal trends in anaesthetic technique and reported indications for general anaesthesia across OBAAMA audit cycles are illustrated in Fig. 1. A marked reduction in general anaesthesia rates occurred between 2011 and 2015, followed by a plateau between 2015 and 2022. This pattern was observed for both elective and emergency caesarean sections. Across audit cycles, maternal preference remained the dominant reported indication for elective general anaesthesia, accounting for approximately 60% of cases in 2022 in both countries. In emergency caesarean sections, urgency remained the predominant reported indication for general anaesthesia.

### Reasons for general anaesthesia

Reported indications for general anaesthesia are summarised in Table 2. In elective caesarean sections, maternal preference was the leading indication in both countries. In emergency procedures, urgency was the predominant reported reason.

**Table 2:** Population-weighted estimates of the reason for administering general anaesthesia

	Czech Republic		Slovakia	
	Elective CS	Emergency CS	Elective CS	Emergency CS
	*N* = 225*	*N* = 508*	*N* = 173**	*N* = 237**
Urgency	0.4% (0.4–0.4)	70.2% (67.3–73.0)	6.1% (2.3–9.9)	85.5% (80.9–90.2)
Maternal preference	62.9% (57.8–68.0)	14.4% (12.2–16.6)	60.8% (53.6–68.1)	6.4% (3.0–9.7)
LMWH applied	3.1% (0.8–5.4)	1.1% (0.4–1.9)	0.7% (0.2–1.1)	1.9% (0.0–3.9)
Bleeding complications	1.1% (0.0–2.8)	1.8% (0.8–2.9)	2.3% (0.1–4.5)	1.4% (0.0–2.9)
Failure of neuraxial blockade	14.5% (11.4–17.6)	4.9% (4.2–5.5)	15.9% (10.6–21.2)	2.8% (0.5–5.1)
Placentation disorder	1.3% (0.8–1.6)	2.1% (1.0–3.1)	0.0%	0.0%
Other	16.6% (12.7–20.6)	5.6% (4.3–6.9)	14.2% (9.0–19.4)	1.9% (0.4–3.4)

Data are given as percentages (95% CI). National numbers of caesarean deliveries were derived from official statistics of the Institute of Health Information of the Czech Republic (*) and the National Health Care Information Centre of Slovakia (**). Identical confidence interval limits indicate zero sampling variance in strata with 100% participation after finite population correction. Emergency caesarean deliveries include urgency categories 1–3 according to the widely used Lucas classification [10]. *CS* caesarean section, *LMWH* low molecular weight heparin

Across audit cycles, maternal preference consistently represented the leading indication for elective general anaesthesia (Czech Republic: 64.1% in 2011, 67.2% in 2015, and 62.9% in 2022; Slovakia: 56.8% in 2015 and 60.8% in 2022).

### Characteristics of general anaesthesia

Rapid sequence induction with tracheal intubation was used in most general anaesthesia cases. Suxamethonium was the preferred neuromuscular blocking agent, and sevoflurane was the predominant volatile anaesthetic. Propofol was used slightly more frequently than thiopental for induction. Difficult intubation was reported in approximately 2% of cases. No cases of pulmonary aspiration were recorded during the study period. Detailed information on positioning, airway management and induction agents is provided in Table 3.

**Table 3:** General anaesthesia–characteristics of elective and emergency caesarean sections

	Czech Republic		Slovakia	
	Elective CS	Emergency CS	Elective CS	Emergency CS
	*N* = 154	*N* = 383	*N* = 89	*N* = 127
Positioning before induction to general anaesthesia				
Anti-Trendelenburg position	17.7%	15.9%	14.6%	15.0%
Tilted to the left side	20.6%	21.0%	15.9%	20.8%
No positioning/supine	61.7%	63.1%	69.5%	64.2%
Airway management				
Rapid sequence induction	87.8%	96.5%	96.6%	98.4%
Sellick’s manoeuvre	10.6%	28.1%	4.9%	34.0%
Orotracheal intubation	99.4%	99.5%	98.9%	100.0%
Laryngeal mask	0.6%	0.5%	1.1%	0
Airway management—complications				
Difficult intubation	2.6%	1.6%	2.2%	0.8%
Regurgitation	0	0.8%	0	0.8%
Aspiration	0	0	0	0
Desaturation (< 90%)	3.2%	0.5%	0	0
Other	1.3%	0	0	0
Induction to anaesthesia				
Thiopental	41.6%	37.6%	24.7%	32.3%
Propofol	53.9%	60.8%	75.3%	66.1%
Etomidate	1.3%	0.3%	0	0
Other/unspecified	3.2%	1.3%	0	0
Suxamethonium	94.2%	97.4%	94.4%	95.3%
Rocuronium	3.6%	2.3%	2.2%	3.1%
Other/unspecified	2.2%	0.3%	3.4%	1.6%
Sufentanil	5.8%	7.6%	3.4%	6.3%
Remifentanil	0	0	0	0.8%
Ketamine	4.5%	5.7%	1.1%	0
Inhalation anaesthetics				
None	5.2%	6.0%	11.2%	22.8%
Nitrous oxide	31.8%	33.2%	23.6%	17.3%
Sevoflurane	85.7%	90.6%	74.2%	88.2%
Desflurane	2.6%	1.3%	0	0

Data are in %. Emergency caesarean deliveries include urgency categories 1–3 according to the widely used Lucas classification [10]. Categories are not mutually exclusive; inhalational agents may have been used in combination. *CS* caesarean section

### Characteristics of spinal anaesthesia

Technical characteristics of spinal anaesthesia are presented in Table 4. Spinal anaesthesia was predominantly performed as a single-shot technique in both elective and emergency settings.

**Table 4:** Spinal anaesthesia–characteristics of elective and emergency caesarean sections

	Czech Republic		Slovakia	
	Elective CS	Emergency CS	Elective CS	Emergency CS
	*N* = 537	*N* = 352	*N* = 251	*N* = 188
Position for spinal puncture				
Lateral	20.1%	23.3%	2.8%	2.1%
Sitting	76.4%	72.2%	90.4%	91.5%
Height of the blockade application				
L1/L2	1.1%	1.4%	0.8%	1.1%
L2/L3	25.7%	17.3%	18.7%	19.7%
L3/L4	62.8%	69.9%	74.1%	70.2%
L4/L5	10.2%	11.4%	6.4%	9.0%
L5/S1	0.2%	0	0	0
Needle				
25 G	18.2%	20.0%	43.6%	38.7%
26 G	48.9%	45.1%	27.8%	23.7%
27 G	32.9%	34.9%	28.6%	37.6%
Introducer used	89.8%	88.9%	89.6%	86.7%
Needle tip				
Quincke	25.6%	33.7%	47.2%	53.4%
Pencil point	48.6%	53.0%	38.7%	36.2%
Atraucan	24.2%	12.1%	14.0%	10.3%
Other	1.6%	1.2%	0	0
Spinal mixture				
Bupivacaine 0.5%	88.3%	84.4%	94.8%	91.0%
– dose (ml)	2.60 [2.40–2.80]	2.60 [2.50–2.80]	2.50 [2.20–2.60]	2.50 [2.20–2.70]
Levobupivacaine 0.5%	*10.8%*	*15.1%*	*4.4%*	*10.1%*
– dose (ml)	*2.80 [2.50–3.00]*	*2.80 [2.60–3.00]*	*2.70 [2.50–2.95]*	*3.00 [2.50–3.00]*
Other/unspecified	0.9%	0.5%	0.8%	
Adjuvants (intrathecal)				
Morphine	24.4%	24.4%	0	0
Fentanyl	0	0	2.0%	3.7%
Alpha-agonist	0	0	0	0
Adrenaline	0	0	0	0
Bicarbonate	0	0	0	0
Position after puncture				
Back	52.4%	57.7%	60.4%	58.2%
Left tilt	46.5%	39.5%	38.7%	40.3%
Insufficiency of anaesthesia				
Pain	1.3%	1.2%	0.4%	1.6%
Restlessness	2.8%	4.8%	0.8%	0.5%
Discomfort	6.0%	5.4%	0.8%	0.6%
Systemic adjuvants for insufficient block				
Propofol	16.7%	3.7%	40.0%	60.0%
Thiopental	1.9%	1.9%	0	0
Midazolam	35.2%	47.5%	60.0%	20.0%
Diazepam	22.2%	25.0%	0	0
Nitrous oxide	1.9%	0	0	20.0%
Sevoflurane	3.7%	0	20.0%	20.0%
Sufentanil	46.3%	30.0%	60.0%	60.0%
Alfentanil	0	0	0	0
Fentanyl	0	0	0	0
Remifentanil	0	0	0	0

Data are given in % or median [IQR]. Percentages for “Insufficiency of anaesthesia” refer to all spinal anaesthesia cases. Subcategories (pain, restlessness, discomfort) are conditional percentages calculated among cases with insufficient block. Bupivacaine 0.5% refers to hyperbaric bupivacaine. If isobaric bupivacaine was used, it was recorded under “Other/unspecified”. Due to small numbers, percentages should be interpreted cautiously. Emergency caesarean deliveries include urgency categories 1–3 according to the widely used Lucas classification [10]. *CS* caesarean section

Patient positioning, needle type selection, and the use of intrathecal adjuvants varied between the countries. The reported rate of failed or insufficient block was low in both countries.

### Characteristics of epidural anaesthesia

Technical characteristics of epidural anaesthesia, including de novo placement and labour epidural top-up conversion, are summarised in Additional file 1: Table S3. Epidural techniques were used either electively as primary anaesthetic procedures or in urgent settings as extensions of labour analgesia, with distinct practice patterns in each country.

### Supplementary material

Additional detailed data on national representativeness, maternal characteristics, preparation before caesarean section, indications for caesarean delivery, epidural techniques, organisational aspects of anaesthesia care, and neonatal outcomes are provided in Additional file 1. Detailed neonatal data are presented in Additional file 1: Table S6 and should not be interpreted as comparative outcome measures between anaesthetic techniques, given the substantial confounding by indication and procedural urgency inherent in this observational design.

## Discussion

Our binational audit shows that general anaesthesia remains disproportionately used for caesarean section in both the Czech Republic and Slovakia, accounting for approximately one-third of all procedures and one-quarter of elective cases. After the marked reduction observed between 2011 and 2015 [6, 7], current data indicate stagnation rather than further decline. Elective general anaesthesia rates of 23–25% contrast with reported Western European rates below 1% [5]. This divergence is clinically relevant because contemporary guidelines and European consensus recommendations define neuraxial anaesthesia as the preferred technique for caesarean section whenever feasible and emphasise the importance of structured obstetric anaesthesia services, including timely access to neuraxial analgesia and anaesthesia [3, 4, 12]. Medical contraindications to neuraxial techniques accounted for only a minority of cases, highlighting a persistent gap between international recommendations and Central European clinical practice.

### Socio-cultural barriers and maternal preference

The leading reported contributor to elective general anaesthesia was maternal preference, which has remained remarkably stable across audit cycles, accounting for approximately 60% of elective general anaesthesia cases over more than a decade [6, 7]. This temporal stability suggests a multifactorial, deeply embedded regional pattern rather than a simple failure of antenatal preparation, inadequate counselling, or a lack of anaesthesiologist expertise. Although qualitative data from Central Europe are lacking, studies from other settings indicate that preference for general rather than neuraxial anaesthesia may be influenced by preoperative anxiety, fear of intraoperative pain or being awake during surgery, concerns about neuraxial needle placement, back pain, spinal cord injury or paralysis, previous negative obstetric or anaesthetic experience, psychiatric comorbidity, and non-medical sources of information [13–17]. Maternal preference should therefore be interpreted not as a single causal category, but as a complex construct shaped by psychological, experiential, informational, and sociocultural determinants. Importantly, this pattern should not be regarded as an optimal standard, but as a persistent phenomenon requiring structured multidisciplinary antenatal counselling and future qualitative research. In this context, anaesthesiologist involvement should occur early enough to support informed consent, address misconceptions about neuraxial techniques and general anaesthesia, and distinguish persistent informed preference from modifiable fear or misinformation.

### Emergency caesarean sections, urgency, and neuraxial infrastructure

In emergency procedures, “urgency” was the predominant reported reason for general anaesthesia. However, this finding requires cautious interpretation. Emergency caesarean sections in this audit included Lucas categories 1–3 [10, 11], and the indication “urgency” reflected real-world documentation by the attending anaesthesiologist rather than externally adjudicated criteria. It therefore does not necessarily correspond to an objective category 1 caesarean section requiring immediate delivery under general anaesthesia.

A key systemic barrier in both countries is the absence of a uniformly implemented national urgency classification. In routine practice, the obstetric team usually declares the urgency of delivery, whereas the anaesthesiologist remains responsible for selecting and safely administering the anaesthetic technique. Without an objective urgency framework, the anaesthesiologist may have limited practical ability to challenge whether sufficient time exists for a de novo neuraxial block, particularly under high-stress team conditions. This decision-making asymmetry may contribute to general anaesthesia being chosen even in some cases where neuraxial anaesthesia might have been feasible with clearer urgency stratification and multidisciplinary communication.

This issue is amplified by the low utilisation of labour epidural analgesia, reported as 20% in the Czech Republic and 30% in Slovakia [8]. European data show substantial variation in labour epidural use across countries, with several Western and Northern European systems reporting substantially higher rates [18]. This difference is important for caesarean section practice: in systems with high labour epidural utilisation, a functioning epidural catheter is often already in place and can be rapidly extended to surgical anaesthesia. In contrast, when no catheter is available, the anaesthesiologist must choose between de novo spinal anaesthesia under perceived time pressure and general anaesthesia. Thus, limited labour epidural infrastructure may contribute to higher general anaesthesia use in emergency caesarean sections independently of anaesthesiologists’ technical ability to perform neuraxial anaesthesia.

Labour epidural underuse should not be viewed as a uniform national cultural phenomenon, because marked heterogeneity exists between individual maternity units and is not explained simply by hospital size or level of perinatal care. Local staffing models, availability of a dedicated 24/7 epidural service, engagement of obstetricians and midwives, persistent outdated concerns regarding labour prolongation or caesarean delivery despite contrary evidence [19], and non-medical narratives favouring unmedicated birth may all contribute. These organisational factors are directly relevant to the European minimum standards for obstetric anaesthesia services, which emphasise reliable access to neuraxial techniques and structured departmental organisation [4].

### Pharmacological practice and postoperative recovery

The audit also identified pharmacological and organisational barriers that may reduce the full benefit of neuraxial techniques. In epidural anaesthesia, adrenaline was not consistently recorded as part of the local anaesthetic mixture. Its absence may reduce the reliability, duration, and quality of epidural extension and could contribute to insufficient anaesthesia or conversion to general anaesthesia. As this was an audit, we cannot determine whether this reflected deliberate practice, incomplete documentation of the epidural mixture, or local protocol variability. Nevertheless, standardisation of epidural top-up regimens represents a relevant target for improvement.

The low use of intrathecal morphine is another important finding. Long-acting neuraxial opioids are a key component of contemporary post-caesarean analgesia and Enhanced Recovery After Caesarean Delivery pathways [20, 21]. In our region, preservative-free morphine suitable for intrathecal administration is not commercially available in routine clinical practice. In the Czech Republic, it can be prepared only as a magistral formulation by selected hospital pharmacies, predominantly in university hospitals, and its availability for other maternity units is limited. This restricted access may reduce one of the major advantages of spinal anaesthesia for caesarean section: simple, effective, opioid-sparing postoperative analgesia that supports early mobilisation, maternal comfort, breastfeeding, and early maternal–newborn bonding. Wider implementation of high-quality neuraxial anaesthesia should therefore be viewed not only as a strategy to reduce exposure to general anaesthesia, but also as a pathway to improve postoperative Quality of Recovery and maternal experience.

These patient-centred postoperative outcomes were not captured in the present audit. Length of stay, opioid consumption, postoperative pain trajectories, maternal satisfaction, breastfeeding, validated Quality of Recovery measures, ERAC-related endpoints, and early bonding should therefore be included in future studies. A dedicated follow-up analysis focusing on determinants of general anaesthesia use and patient-centred postoperative outcomes is currently being planned.

### Safety profile and neonatal outcomes

Neuraxial anaesthesia remains the preferred technique for caesarean section whenever feasible, because it avoids airway manipulation and provides important benefits for postoperative analgesia, maternal experience, and recovery. However, historical mortality estimates comparing general and neuraxial anaesthesia should be interpreted cautiously in contemporary practice. The often-cited excess maternal mortality associated with general anaesthesia is largely derived from historical observational data collected before current obstetric airway algorithms, the routine availability of supraglottic rescue devices, the wider use of videolaryngoscopy, and contemporary monitoring standards [22, 23]. Moreover, such comparisons are highly vulnerable to confounding by indication, because general anaesthesia is preferentially used in the most urgent and highest-risk cases. Our audit was not designed to estimate causal differences in maternal morbidity or mortality between anaesthetic techniques. Therefore, the aim should not be to frame contemporary general anaesthesia as uniformly unsafe, but to reduce potentially avoidable general anaesthesia through better counselling, neuraxial infrastructure, urgency classification, and team communication, while maintaining the ability to deliver safe general anaesthesia when clinically indicated or strongly requested after informed counselling.

In our cohort, airway management was consistent with contemporary obstetric standards [24]. Rapid sequence induction with tracheal intubation was used in most general anaesthesia cases; difficult intubation occurred in approximately 2%, and no aspiration events were recorded. These findings suggest a high degree of airway safety, but should be interpreted with the limitations of registry-based reporting and the short audit period in mind.

Neonatal outcomes are presented in Additional file 1: Table S6. Lower Apgar scores at 1 and 5 min were observed in the general anaesthesia group; however, these differences should be interpreted descriptively and not as causal effects of anaesthetic technique. General anaesthesia was used more frequently in emergency and higher-risk procedures, creating substantial confounding by urgency, indication, and underlying foetal status [25]. Umbilical arterial pH values were comparable between anaesthetic techniques, and Apgar scores largely improved by 10 min in both groups. These findings are consistent with transient neonatal depression and differences in case mix rather than demonstrating clinically meaningful hypoxic injury attributable to general anaesthesia. Because the audit was not designed or powered as a neonatal outcome study, these data should be considered hypothesis-generating only.

Financial incentives are unlikely to explain the observed pattern. In both the Czech Republic and Slovakia, there are no direct payments to obstetricians, anaesthesiologists, or institutions based on the type of anaesthesia used for caesarean section. Anaesthetic care is reimbursed as part of the broader DRG-based hospital payment system; therefore, there is no direct financial incentive favouring general anaesthesia over neuraxial techniques.

### Strengths and limitations

The main strength of this study is its prospective binational audit design combined with population weighting based on official national statistics [9]. This approach mitigates selection bias inherent in voluntary surveys and generates representative national estimates. Alignment with previous OBAAMA audits further permits a unique longitudinal comparison over more than a decade [6, 7].

Several limitations must be acknowledged. The observational and descriptive design precludes risk adjustment and causal inference. Reported reasons for general anaesthesia were selected by attending anaesthesiologists from predefined registry categories and were not externally adjudicated. Urgency categories were analysed as a single aggregate group, and no case-level data were collected on decision-to-delivery interval, time of day, or individual provider seniority; therefore, the contribution of out-of-hours practice, anaesthesiologist experience, and obstetrician experience could not be directly assessed. The audit also did not collect qualitative data on maternal preferences, antenatal counselling, decision-making processes, or local reasons for low epidural utilisation during labour. Neonatal outcomes were reported descriptively and were not adjusted for urgency, indication, or foetal status. While population weighting was applied, voluntary participation may still introduce residual selection bias, particularly given Slovakia's lower participation rate. Finally, the one-month audit period limited the capture of rare complications and patient-centred postoperative outcomes.

## Conclusion

These population-weighted data show that contemporary anaesthetic practice for caesarean section in the Czech Republic and Slovakia continues to differ substantially from recommended European standards. General anaesthesia remains common, particularly in elective cases, attributed to maternal preference and in emergency cases, attributed to urgency. These findings should not be interpreted as reflecting a single cause, but rather a stable interaction between sociocultural expectations, antenatal counselling pathways, labour epidural availability, non-standardised urgency classification, interdisciplinary decision-making under time pressure, and regional pharmacological and organisational constraints.

Reducing potentially avoidable general anaesthesia will require structured antenatal counselling with earlier anaesthesiologist involvement, expansion of labour epidural infrastructure in line with European obstetric anaesthesia service standards [4], standardised epidural top-up protocols, improved access to neuraxial opioid analgesia, nationally accepted urgency classification, and multidisciplinary communication pathways that allow anaesthesiologists to make technique decisions within an objective and shared framework.

## Supplementary Information

Additional file 1: Table S1. Caesarean sections and anaesthesia used in the Czech Republic and Slovakia, comparison of OBAAMA-COV data and national weighted estimates. Table S2. Basic characteristics of women who have undergone a caesarean section. Table S3. Epidural anaesthesia – characteristics of elective and emergency caesarean sections. Table S4. Basic preparation of the mother before a caesarean section. Table S5. Indications for caesarean section according to the type of anaesthesia used. Table S6. Neonatal data (*weighted estimates*). Table S7. Characteristics of anaesthesia teams in Czech and Slovak maternity hospitals (*weighted estimate*).

## Data Availability

The datasets used and/or analysed during the current study are available from the corresponding author on reasonable request.

## Abbreviations

OBAAMA: Obstetric Anaesthesia and Analgesia Attributes
CS: Caesarean section
CSE: Combined spinal-epidural anaesthesia
CSARIM: Czech Society for Anaesthesiology, Resuscitation, and Intensive Care Medicine
SSAIM: Slovak Society of Anaesthesiology and Intensive Medicine
ESAIC: European Society of Anaesthesiology and Intensive Care
HELLP: Haemolysis, Elevated Liver enzymes and Low Platelet count
LMWH: Low molecular weight heparin
ÚZIS: Institute of Health Information and Statistics of the Czech Republic
NCZI: National Health Information Centre of Slovakia
CI: Confidence intervals
IQR: Interquartile ranges
