# Supplementary material for: Obstetric anaesthesia for caesarean section in Central Europe: a prospective population-weighted binational audit (OBAAMA-COV Study)
Source: J Anesth Analg Crit Care. 2026 Jul 8;6:122. doi: 10.1186/s44158-026-00435-2 (PMC13628821; doi:10.1186/s44158-026-00435-2)
Supplement: Supplementary file 1 [file 44158_2026_435_MOESM1_ESM.docx]

**ADDITIONAL FILE 1:**

**TABLE S1**. Caesarean sections and anaesthesia used in the Czech Republic and Slovakia, comparison of OBAAMA-COV data and national weighted estimates.

**TABLE S2**. Basic characteristics of women who have undergone a caesarean section.

**TABLE S3**. Epidural anaesthesia – characteristics of elective and emergency caesarean sections.

**TABLE S4**. Basic preparation of the mother before a caesarean section.

**TABLE S5**. Indications for caesarean section according to the type of anaesthesia used.

**TABLE S6**. Neonatal data (*weighted estimates*).

**TABLE S7**. Characteristics of anaesthesia teams in Czech and Slovak maternity hospitals (*weighted estimate*).

**Table S1. Caesarean sections and anaesthesia used in the Czech Republic and Slovakia, comparison of OBAAMA-COV data and national weighted estimates.**

|  | **CZECH REPUBLIC** (OBAAMA-COV) | | **SLOVAKIA**  (OBAAMA-COV) | | **CZECH REPUBLIC** (weighted estimate) | | **SLOVAKIA**  (weighted estimate) | |
| --- | --- | --- | --- | --- | --- | --- | --- | --- |
| **Number of births** | 5914 | | 2030 | | 7749* | | 3490** | |
| **Number of CS** | 1581 (26.7%) | | 689 (33.9%) | | 2068 (26.7%)* | | 1205 (34.5%)** | |
| **General anaesthesia** | 537 (34.0%) | | 216 (31.3%) | | 33.9% | | 31.3% | |
| **Neuraxial anaesthesia** | 1044 (66.0%) | | 473 (68.7%) | | 66.1% | | 68.7% | |
|  | **Elective CS** | **Emergency CS** | **Elective CS** | **Emergency CS** | **Elective CS** | **Emergency CS** | **Elective CS** | **Emergency CS** |
|  | *717 (45.4%)* | *864 (54.6%)* | *345 (50.1%)* | *344 (49.9%)* | *940** *(45.5%)* | *1128** *(54.5%)* | *606** *(50.3%)* | *599** *(49.7%)* |
| General anaesthesia | 154 (21.5%) | 383 (44.3%) | 89 (25.8%) | 127 (36.9%) | 23.0% (19.9–26.4) | 45.4% (42.1–48.8) | 24.9%  (20.6–29.7) | 36.8%  (31.8–42.1) |
| Neuraxial anaesthesia | 563 (78.5%) | 481 (55.7%) | 256 (74.2%) | 217 (63.1%) | 77.0% (73.6–80.1) | 54.6% (51.2–57.9) | 75.1%  (70.3–79.4) | 63.2%  (57.9–68.2) |

*Data are given in numbers (%); the weighted estimate is given with a 95% confidence interval (CI); national numbers of caesarean deliveries were derived from official statistics of the Institute of Health Information of the Czech Republic (*) and the National Health Care Information Centre of Slovakia (**). Emergency caesarean deliveries include urgency categories 1–3 according to the widely used Lucas classification [10]. CS–caesarean section.*

**Table S2. Basic characteristics of women who have undergone a caesarean section.**

|  | **CZECH REPUBLIC** (OBAAMA-COV) | **SLOVAKIA**  (OBAAMA-COV) | **CZECH REPUBLIC** (weighted estimate) | **SLOVAKIA**  (weighted estimate) |
| --- | --- | --- | --- | --- |
|  | N = 1581 | N = 689 | N=2068 | N=1205 |
| **Age of mother (years)** | 32 [28–35] | 31 [27–35] | 32 (31–33) | 31 (27-35) |
| **Current weight (kg)** | 81 [72–93] | 78 [68–90] | 81 (80–82) | 78 (77–80) |
| **Height (cm)** | 166 [161–170] | 165 [160–170] | 166 (166–167) | 165 (165–167) |
| **BMI** | 29.4 [24.9–35.9] | 28.7 [23.5–35.2] | 29.4 (28.7–29.8) | 28.7 (27.6–29.4) |
| **Week of gestation** |  |  |  |  |
| 23–36 | 13.9% | 12.3% | 14.1% (12.4–16.0) | 11.9% (9.72–14.5) |
| 37–40 | 71.6% | 73.7% | 71.7% (69.3–73.9) | 74.0% (70.6–77.1) |
| >40 | 14.4% | 14.3% | 14.2% (12.6–16.1) | 14.1% (11.7–16.9) |
| **Induced labour** | 17.6% | 14.9% | 17.7% (15.8–19.7) | 14.9% (12.4–17.8) |
| **Gravidity** |  |  |  |  |
| 1 | 43.3% | 43.7% | 43.1% (40.6–45.6%) | 43.5% (39.8–47.2%) |
| 2 | 34.6% | 32.9% | 34.7% (32.4–37.2) | 33.0% (29.6–36.6) |
| >2 | 22.1% | 23.4% | 22.2% (20.1–24.3) | 23.5% (20.5–26.9) |
| **ASA** |  |  |  |  |
| 1 | 42.7% | 41.7% | 43.2% (40.7–45.7) | 42.1% (38.4–45.9) |
| 2 | 50.0% | 55.3% | 50.2% (47.6–52.7) | 55.0% (51.2–58.7) |
| 3 | 6.4% | 3.0% | 5.7% (4.74–6.96) | 2.9% (1.92–4.48) |
| 4 | 0.8% | 0 | 0.7% (0.41–1.28) | – |
| 5 | 0.2% | 0 | 0.2% (0.06–0.54) | – |
| **Coagulation disorder** | 4.4% | 4.2% | 4.6% (3.61–5.78) | 4.3% (2.98–6.10) |
| **Thrombocytopenia** | 1.2% | 0.9% | 1.3% (0.84–2.11) | 0.9% (0.38–1.90) |
| **Neurological diseases** | 4.7% | 5.6% | 4.91% (3.86–6.17) | 5.73% (3.97–8.13) |
| **Gestational diabetes** | 14.6% | 6.4% | 14.5% (12.8–16.4) | 6.4% (4.75–8.45) |
| **Hypertension of pregnancy** | 6.8% | 5.5% | 7.0% (5.82–8.47) | 5.4% (3.95–7.36) |
| **Preeclampsia** | 4.5% | 6.2% | 4.5% (3.55–5.68) | 6.3% (4.71–8.43) |
| **Eclampsia** | 0.1% | 0 | 0.1% (0.03–0.48) | – |
| **HELLP** | 0.4% | 0.6% | 0.4% (0.21–0.93) | 0.5% (0.20–1.43) |
| **Placentation disorder** | 2.4% | 1.0% | 2.4% (1.76–3.39) | 1.0% (0.48–2.13) |
| **COVID-19 positive** | 0.4% | 1.0% | 0.4% (0.18–0.90) | 1.1% (0.50–2.22) |

*Data are presented as %, numbers (95% CI), or median [IQR]. ASA–American Society of Anaesthesiologists Physical Status Classification System, HELLP–Haemolysis, Elevated Liver Enzymes, Low Platelets Syndrome.*

**Table S3. Epidural anaesthesia – characteristics of elective and emergency caesarean sections.**

|  | **CZECH REPUBLIC** | **SLOVAKIA** |
| --- | --- | --- |
|  | N=166 | N = 36 |
| **Introduced "de novo"** | 66 | 3 |
| **Top-up anaesthesia** | 100 | 29 |
| **Position for epidural puncture** |  |  |
| - Lateral | 69.2% | 22.6% |
| - Sitting | 30.8% | 77.7% |
| **Height of the blockade application** |  |  |
| Th12/L1 | 3.6% | 0 |
| L1/L2 | 26.5% | 0 |
| L2/L3 | 54.8% | 44.8% |
| L3/L4 | 15.1% | 48.3% |
| L4/L5 | 0 | 6.9% |
| **Needle** |  |  |
| Tuohy 18 G | 100% | 100% |
| Catheter G20 | 87.7% | 90.0% |
| Catheter G19 | 12.3% | 10.0% |
| **Identification of the epidural space** |  |  |
| Hanging drop | 3.4% | 0 |
| Loss of resistance | 91.1% | 96.7% |
| A feeling of penetration through the yellow ligament with a needle | 5.5% | 3.3% |
| **Applied a local anaesthetic** |  |  |
| Bupivacaine 0.5% | 53.0% | 5.6% |
| Levobupivacaine 0.5% | 1.2% | 66.7% |
| Lidocaine 2% | 45.8% | 27.7% |
| **Adjuvants to local anaesthetic** |  |  |
| Sufentanil | 83.7% | 94.4% |
| Fentanyl | 0 | 0 |
| Alpha-agonist | 0 | 0 |
| Adrenaline | 51.3% | 33.3% |

*Data are given in n or %. LA–local anaesthetics. Epidural top-up refers to the extension of labour epidural analgesia to anaesthesia for caesarean section; epidural de novo refers to newly administered epidural anaesthesia.*

**Table S4. Basic preparation of the mother before a caesarean section.**

|  | **CZECH REPUBLIC** | | **SLOVAKIA** | |
| --- | --- | --- | --- | --- |
|  | **Elective CS** | **Emergency CS** | **Elective CS** | **Emergency CS** |
|  | N = 717 | N = 864 | N = 345 | N = 344 |
| **Prevention of aspiration** |  |  |  |  |
| No prevention | 35.3% | 58.3% | 20.3% | 18.3% |
| H_2_ blocker | 22.2% | 6.8% | 34.2% | 25.6% |
| Metoclopramide | 43.9% | 28.2% | 59.1% | 57.6% |
| Proton pump inhibitor | 17.4% | 6.5% | 19.1% | 23.3% |
| 0.3M sodium citrate | 0.7% | 0.6% | 55.9% | 63.4% |
| 0.1M sodium citrate | 0 | 0 | 0.6% | 0.3% |
|  |  |  |  |  |
| **Mallampati Score** |  |  |  |  |
| I | 38.6% | 30.6% | 40.1% | 38.9% |
| II | 50.5% | 54.5% | 52.6% | 53.6% |
| III | 9.7% | 12.6% | 6.3% | 6.8% |
| IV | 1.2% | 2.2% | 1.0% | 0.7% |

*Data are given in n or %.*

**Table S5. Indications for caesarean section according to the type of anaesthesia used.**

|  | **CZECH REPUBLIC** | | | | **SLOVAKIA** | | | |
| --- | --- | --- | --- | --- | --- | --- | --- | --- |
|  | **Elective CS** | | **Emergency CS** | | **Elective CS** | | **Emergency CS** | |
|  | N = 154 | N = 383 | N = 563 | N = 481 | N = 89 | N = 256 | N = 127 | N = 217 |
| **Indications for caesarean section** | General anaesthesia | Neuraxial anaesthesia | General anaesthesia | Neuraxial anaesthesia | General anaesthesia | Neuraxial anaesthesia | General anaesthesia | Neuraxial anaesthesia |
| Previous CS | 35.7% | 41.6% | – | – | 37.1% | 44.9% | – | – |
| Breech position | 13.0% | 16.2% | – | – | 9.0% | 13.3% | – | – |
| Cephalopelvic disproportion | 5.8% | 6.7% | – | – | 7.9% | 9.0% | – | – |
| Maternal preference | 5.2% | 5.0% | – | – | 3.4% | 1.2% | – | – |
| Twins | 3.2% | 5.2% | – | – | 9.0% | 3.9% | – | – |
| Neurological diseases | 9.1% | 1.4% | – | – | 14.6% | 2.3% | – | – |
| Internal diseases | 2.6% | 3.0% | – | – | 3.4% | 6.3% | – | – |
| Placentation disorder | 1.9% | 3.6% | – | – | 0 | 2.0% | – | – |
| Eye diseases | 0.6% | 1.4% | – | – | 1.1% | 1.6% | – | – |
| Other | 22.7% | 16.0% | – | – | 14.6% | 15.6% | – | – |
| Non-progressive childbirth | – | – | 17.5% | 44.7% | – | – | 24.4% | 40.1% |
| Signs of foetal hypoxia | – | – | 47.3% | 20.4% | – | – | 40.2% | 23.5% |
| Elective CS in labour | – | – | 8.4% | 17.0% | – | – | 6.3% | 14.3% |
| Preeclampsia | – | – | 3.7% | 3.3% | – | – | 7.9% | 5.5% |
| Placental abruption | – | – | 6.5% | 1.0% | – | – | 7.1% | 0.9% |
| Other bleeding | – | – | 2.9% | 0.2% | – | – | 2.4% | 0.5% |
| Other | – | – | 13.8% | 13.3% | – | – | 11.8% | 15.2% |

*Data are given in %. Emergency caesarean deliveries include urgency categories 1–3 according to the widely used Lucas classification [10]. CS–caesarean section.*

**Table S6. Neonatal data (weighted estimates).**

|  | **CZECH REPUBLIC** | | **SLOVAKIA** | |
| --- | --- | --- | --- | --- |
| Number of children born | 2191* | | 789** | |
| **Birth weight (g)** | 3250 [3220–3300] | | 3300 [3250–3350] | |
| **Sex of the newborn** | | | | |
| Male | 48.5% (44.6–52.4) | | 57.3% (40.1–72.9) | |
| Female | 51.5% (47.6–55.4) | | 42.7% (27.1–59.9) | |
|  | **General anaesthesia** | **Neuraxial anaesthesia** | **General anaesthesia** | **Neuraxial anaesthesia** |
| **pH of arterial umbilical cord blood** | 7.31 (7.27–7.36) | 7.32 (7.29–7.36) | 7.32 (7.29–7.35) | 7.33 (7.28–7.36) |
| **Apgar score 1 min** | | | | |
| **0–4** | 8.6% (6.8–11.0) | 2.4% (1.9–3.1) | 4.9% (2.9–8.0) | 1.3% (0.8–2.1) |
| **5–7** | 12.2% (10.3–14.3) | 6.4% (5.4–7.5) | 8.9% (6.6–12.2) | 4.0% (3.0–5.5) |
| **8–10** | 79.2% (77.5–80.8) | 91.2% (89.2–93.0) | 86.2% (83.9–87.9) | 94.7% (92.2–96.6) |
| **Apgar score 5 min** | | | | |
| **0–4** | 2.4% (1.4–4.1) | 0.2% (0.1–0.3) | 1.8% (0.7–4.1) | 0.4% (0.2–0.9) |
| **5–7** | 7.0% (5.5–8.8) | 2.8% (2.2–3.5) | 4.5% (2.8–7.4) | 1.9% (1.2–3.1) |
| **8–10** | 90.6% (89.4–91.7) | 97.0% (95.7–98.1) | 93.7% (92.2–94.8) | 97.7% (96.1–98.8) |
| **Apgar score 10 min** | | | | |
| **0–4** | 0.9% (0.4–2.2) | 0 (0.0–0.0) | 1.4% (0.6–3.4) | 0.9% (0.4–2.2) |
| **5–7** | 1.9% (1.2–3.0) | 1.0% (0.6–1.6) | 1.1% (0.4–3.7) | 0.3% (0.1–1.0) |
| **8–10** | 97.2% (96.4–97.8) | 99.0% (98.2–99.6) | 97.5% (96.1–98.3) | 98.8% (97.4–99.6) |

*Data are presented as counts, median [IQR], or percentages with 95% confidence intervals (CI). Data are not adjusted for urgency or case mix. National numbers of caesarean deliveries were derived from official statistics of the Institute of Health Information of the Czech Republic (*) and the National Health Care Information Centre of Slovakia (**). Neonatal data were collected for descriptive purposes only and should not be interpreted as comparative outcome measures between anaesthetic techniques. CS*–*caesarean section; CI*–*confidence interval.*

**Table S7. Characteristics of anaesthesia teams in Czech and Slovak maternity hospitals *(weighted estimate).***

|  | **CZECH REPUBLIC** | **SLOVAKIA** |
| --- | --- | --- |
| **24/7 mode** | 100.0% | 90.0% (66.9–97.6) |
| **Anaesthesiologists with Specialist Qualification** | 66.6% (61.5–72.2) | 76.9% (75.0–77.1) |
| **Anaesthesiologists in training** | 33.4% (27.8–39.5) | 23.1% (22.9–25.0) |
| **Anaesthesiologist assigned solely to the labour and delivery room** | 78.2% (65.5–87.1) | 89.1% (64.6–97.3) |
| **Father present during CS in neuraxial anaesthesia** | 62.8% (49.6–74.3) | 25.2% (9.84–50.9) |

*Data are given in n (%) or % (95% CI); national numbers of caesarean deliveries were derived from official statistics of the Institute of Health Information of the Czech Republic (*) and the National Health Care Information Centre of Slovakia (**). A Specialist Qualification in Anaesthesiology is equivalent to US Board Certification or a UK Certificate of Completion of Training. Emergency caesarean deliveries include urgency categories 1–3 according to the widely used Lucas classification [10]. CS–caesarean section, CI–confidence interval.*
